# Characterisation of major histocompatibility complex class I genes in Japanese Ranidae frogs

**DOI:** 10.1007/s00251-016-0934-x

**Published:** 2016-07-14

**Authors:** Quintin Lau, Takeshi Igawa, Shohei Komaki, Yoko Satta

**Affiliations:** 1 Department of Evolutionary Studies of Biosystems, Sokendai (The Graduate University for Advanced Studies), Kamiyamaguchi 1560-35, Hayama, Kanagawa 240-0193 Japan; 2 Global Career Design Center, Hiroshima University, 1-7-1, Higashi-Hiroshima, Hiroshima 739-8514 Japan; 3 Division of Developmental Science, Graduate School for International Development and Cooperation, Hiroshima University, 1-5-1, Higashi-Hiroshima, Hiroshima 739-8529 Japan

**Keywords:** Major histocompatibility complex, Selection, *Rana*, Anura

## Abstract

The major histocompatibility complex (MHC) is a key component of adaptive immunity in all jawed vertebrates, and understanding the evolutionary mechanisms that have shaped these genes in amphibians, one of the earliest terrestrial tetrapods, is important. We characterised MHC class I variation in three common Japanese *Rana* species (*Rana japonica*, *Rana ornativentris* and *Rana tagoi tagoi*) and identified a total of 60 variants from 21 individuals. We also found evolutionary signatures of gene duplication, recombination and balancing selection (including trans-species polymorphism), all of which drive increased MHC diversity. A unique feature of MHC class I from these three Ranidae species includes low synonymous differences per site (*d*_S_) within species, which we attribute to a more recent diversification of these sequences or recent gene duplication. The resulting higher *d*_N_/*d*_S_ ratio relative to other anurans studied could be related to stronger selection pressure at peptide binding sites. This is one of the first studies to investigate MHC in Japanese amphibians and permits further exploration of the polygenetic factors associated with resistance to infectious diseases.

**Electronic supplementary material:**

The online version of this article (doi:10.1007/s00251-016-0934-x) contains supplementary material, which is available to authorized users.

## Introduction

Major histocompatibility complex (MHC) genes code for membrane-bound glycoproteins that recognise, bind and present specific antigens to T lymphocytes and, thus, are important components of adaptive immunity in jawed vertebrates. MHC class I molecules bind endogenous antigenic peptides, presenting them to cytotoxic T cells, and comprised of an α heavy chain and β_2_m microglobulin chain. The α chain of MHC class I consists of three extracellular domains (α1, α2 and α3) encoded by exons 2, 3 and 4, respectively. The anchor residues within the peptide binding groove, sometimes referred to as peptide binding sites, are found in the α1 and α2 domains (Hughes and Yeager 1998). There are two groups of class I genes based on functional differences: class Ia (classical) genes which are highly polymorphic and expressed in all nucleated cells and class Ib (non-classical) that are less polymorphic and have variable expression across tissues (Goyos et al. 2011; Hughes and Yeager 1998).

Classical MHC genes are one of the most polymorphic genes of vertebrate genomes (Hedrick 1994), and they evolve under a birth and death process (Nei and Rooney 2005), resulting in variation in MHC loci number between different species. MHC variation is primarily maintained by pathogen-mediated balancing selection (Bernatchez and Landry 2003; Hughes and Yeager 1998). One of the characteristics of balancing selection is trans-species polymorphism, whereby alleles in different species are more closely related than conspecific alleles (Klein et al. 1998), and this has been seen in MHC genes (Wei et al. 2010; Jaratlerdsiri et al. 2014). As the MHC is important for adaptive immunity, several studies in vertebrates have found associations with MHC variation and infectious diseases (summarised by Sommer 2005). In particular, recent studies have identified MHC–disease associations in a number of amphibian species (Bataille et al. 2015; Savage and Zamudio 2011; Teacher et al. 2009).

Anuran amphibians are ectothermic tetrapods with a metamorphic life cycle, comprising a developing pre-metamorphic stage (tadpoles) inhabiting aqueous environments and a post-metamorphic stage occupying terrestrial and/or aquatic habitats. A change in morphology and environment during the life cycle presents the potential for differential exposure to different pathogens during development. There are reports that certain pathogens may have stronger impact on either pre- (Chambouvet et al. 2015; Haislip et al. 2011) or post-metamorphic life stages (Blaustein et al. 2005), supporting that developmental and environmental changes could influence amphibian host immunity. Many amphibian diseases caused by pathogens, including bacteria, viruses, fungi and parasites, have been reported (summarised by Densmore and Green 2007); one of the most commonly documented amphibian infectious diseases in recent times is chytridiomycosis.

Chytridiomycosis is a serious disease in amphibians caused by the chytrid fungus *Batrachochytridium dendrobatidis*, which was identified by Berger et al (1998) and characterised by Longcore et al (1999). Many studies have attributed chytridiomycosis, which causes epidermal disruption, to the decrease in amphibian populations around the world (Daszak et al. 2003; Longcore et al. 2007; Skerratt et al. 2007). In Japan, several strains of *B. dendrobatidis* were characterised as either non-native or apparently endemic strains, although low incidence (4.1 %) was found in free-ranging amphibians (Goka et al. 2009). Some amphibian species in Japan where the fungus was detected include exotic species from the pet trade and the naturalised *Lithobates* (*Rana*) *catesbeianus*, a key carrier species attributed with the spread of this global disease (Garner et al. 2006). It has been suggested that *B. dendrobatidis* is endemic to Asia (Goka et al. 2009; Bataille et al. 2013), implying the coexistence between Japanese (or Asian) amphibians and *B. dendrobatidis* for a long time, which may have allowed the amphibians to evolve an effective resistance against chytrid infection. This could explain the tolerance of endemic amphibians to the fungi and the resulting low incidence in Japan (or Asia). Therefore, initiating the examination of immune genes of Japanese amphibian species will contribute to further understanding amphibian host–disease dynamics.

Within the family Ranidae, MHC class I has been studied in *L. catesbeianus* (American bullfrog), *Lithobates clamitans* (green frog), *Lithobates yavapaiensis* (lowland leopard frog), *Lithobates pipiens* (Northern leopard frog) and *Rana temporaria* (common frog), and signatures of balancing selection have been detected (Flajnik et al. 1999; Kiemnec-Tyburczy et al. 2012; Teacher et al. 2009). Apart from balancing selection, recombination and gene duplication also play a role in driving MHC diversity (Carrington 1999; Yeager and Hughes 1999). Recombination between exons at a single locus has been identified in *Xenopus laevis* (African clawed frog) and two Rhacophoridae species (Bos and Waldman 2006; Zhao et al. 2013). Whilst some model Pipidae and Ranidae species express a single classical MHC class I locus (Flajnik et al. 1999; Goyos et al. 2011; Ohta et al. 2006; Teacher et al. 2009), copy number variation was revealed following research across more expansive families and additional Ranidae species (Kiemnec-Tyburczy et al. 2012; Lillie et al. 2014; Zhao et al. 2013). The aim of this study was to characterise MHC class I genes in three Japanese frog species from the *Rana* genus. In addition, we wanted to investigate whether selective mechanisms and gene duplication have shaped MHC variation. This will contribute to further understanding about the evolution of amphibian MHC class I.

## Methods

### Sample collection and nucleic acid isolation

We selected three *Rana* frog species that are commonly found across Japan: the Japanese brown frog (*Rana japonica*), the montane brown frog (*Rana ornativentris*) and Tago’s brown frog (*Rana tagoi tagoi*). For genetic material, adult individuals (*n* = 7 per species) originating from separate locations within Hiroshima prefecture, Japan, were used: *R. japonica* raised in captivity and originating from multiple egg clusters collected from an island population in Etajima (34°16′14″N, 132°28′37″E); *R. ornativentris* raised in captivity from tadpoles collected from Yoshiwa (34°25′04″N, 132°05′15″E); and *R. t. tagoi* from adult frogs collected from Shobara (34°05′04″N, 132°49′43″E). Adult frogs were euthanized by immersion in tricaine methanesulfonate (MS222, 0.5–3 g/L water) and spleen was collected. Total RNA was extracted using ISOGEN (Nippon Gene, Tokyo, Japan) following the manufacturer’s protocol, and first-strand complementary DNA was synthesised using PrimeScript^TM^ RT reagent kit (Takara Bio Inc., Otsu, Japan).

### MHC class I PCR

Primers were designed to amplify partial coding sequence of MHC class I, spanning from exons 1 to 4, based on transcriptome data (T. Igawa, unpublished data) from skin samples of *R. japonica* as well as several Ranidae species from southern Japanese islands: *Odorrana narina*, *Odorrana amamiensis*, *Odorrana supranarina*, *Odorrana ishikawae*, *Odorrana splendida*, *Babina holsti* and *Babina subaspera*. Based on conserved regions in aligned sequences, we designed primers using Primer3 (Rozen and Skaletsky 1998): forward 5′-GTGTCAGGRGTGKAKTGTG-3′ and reverse 5′-GAAYMTMCTCCAGACTGCTGT-3′, the latter being similar to that of Kiemnec-Tyburczy et al (2012).

Polymerase chain reaction (PCR) amplification was performed in Applied Biosystems® Veriti® thermal cycler in 10 μl reactions containing 0.2 U KOD FX Neo DNA polymerase (Toyobo, Osaka, Japan), 1× PCR buffer, 0.4 mM each dNTP and 0.5 μM each primer. General cycle conditions included initial Taq activation at 95 °C for 1 min, followed by 30 cycles of 30-s denaturation at 95 °C, 30-s annealing at 56 °C and 30-s extension at 72 °C, then a final extension of 72 °C for 3 min. 3′-dA overhangs were then added to PCR products using 10× A-attachment mix (Toyobo), and then the products were ligated into T-Vector pMD20 (Takara Bio Inc.) using DNA Ligation Kit 2.1 (Takara Bio Inc.) and incubated for 16 °C for 30 min. For cloning, ligation reactions were transformed into One Shot® TOP10 competent cells (Invitrogen, Tokyo, Japan). Positive clones (8–16 per individual) were amplified with M13 primers in similar PCR conditions, purified with ExoSAP-IT® (Affymetrix Inc., Santa Clara, USA) and sequenced with BigDye® Terminator Cycle Sequencing Kit (Applied Biosystems, Foster City, USA) and an ABI 3130xl automated sequencer.

### Sequence and phylogenetic analyses

All ABI trace files were edited and analysed using CodonCode Aligner 5.1.5 (CodonCode Co., Centerville, USA). To avoid sequences resulting from PCR or cloning artefacts, we used the following criteria for inclusion of sequences: (1) amplified from more than one clone and (2) differ from other sequences by at least three nucleotides. We allowed for two exceptions where pairs of sequences (*Raja*-UA*06 and 07, and *Raor*-UA*19 and 25) differed by only a single non-synonymous substitution at exon 3 (α2 domain) as they were confirmed by repeat PCR and cloning reactions. For each species, we used MEGA7 (Kumar et al. 2016) to calculate measures of overall mean nucleotide and amino acid distances (*p* distance) across the entire sequence and each domain independently. Using ClustalW, the derived class I amino acid sequences were aligned with those of previously studied amphibian species, including *Ambystoma mexicanum*, *X. laevis*, *Rhacophorus omeimontis*, *Polypedates megacephalus*, *Agalychnis callidryas*, *Espadarana prosoblepon*, *Smilisca phaeota*, *L. catesbeianus*, *L. clamitans* and *L. yavapaiensis* (Flajnik et al. 1999; Kiemnec-Tyburczy et al. 2012; Sammut et al. 1997; Zhao et al. 2013). For phylogenetic analyses, neighbour-joining trees (*p* distance) were constructed from amino acid alignments in MEGA7 using 1000 bootstrap replicates. *Xenopus* non-classical Ib and *Gallus gallus* class I sequences were also included, with the latter used as an outgroup. Trees were constructed separately for α1, α2 and α3 domains. Maximum-likelihood trees (JTT model + G + I as model of best fit, 500 bootstrap replicates) were also constructed in MEGA7 to confirm inferred phylogenetic relationships.

### Tests for recombination and selection

Following Zhao et al (2013), we used two approaches to check for the presence of recombination, briefly: (1) GARD (genetic algorithm recombination detection; Kosakovsky Pond et al. 2006) executed in the online Datamonkey website (http://www.datamonkey.org) and (2) RDP4 (Recombination Detection Program, version 4) which implements multiple methods (Martin et al. 2015). Within RDP4, we only considered breakpoints that were identified by at least four of the seven methods tested (RDP, BOOTSCAN, GENECONV, MAXCHI, CHIMAERA, SISCAN and 3SEQ).

Non-synonymous substitutions per non-synonymous site (*d*_N_) and synonymous substitutions per synonymous site (*d*_S_) were calculated in MEGA7 with the Nei–Gojobori method (proportion). We used codon-based *Z* tests in MEGA7 (10,000 bootstrap replicates, partial deletion) to test for global neutral (*d*_N_ *= d*_S_), positive (*d*_N_ *> d*_S_) and purifying (*d*_N_ *< d*_S_) selections in MHC class I sequences independently for each of the three species. In addition, sequence-wide positive selection was also tested using PARRIS, which is implemented in the Datamonkey website. To test for signatures of positive selection on specific codon sites, we used omegaMap version 5.0 (McVean and Wilson 2006) as well as three methods hosted by the Datamonkey website: fixed effects likelihood (FEL), random effects likelihood (REL) and mixed effects model of evolution (MEME) models. These tests were conducted independently in each of the species as species-specific selection pressures are expected and omegaMap makes the assumption of random mating within all individuals. We used omegaMap to perform Bayesian inference on independent alignments for each species and inferred positively selected codon sites (*ω* or *d*_N_/*d*_S_ > 1) even in the presence of recombination. For each species, we conducted two independent omegaMap runs following the same conditions as Lau et al (2015), but with 1 × 10^6^ MCMC iterations and 100,000 burn-in iterations. We used conservative cutoff probabilities for identification of selected sites; for omegaMap, *ω* > 1 with posterior probability >0.95, FEL and MEME *p* values <0.05 and REL Bayes factor >200. We considered a codon site as being under selection (or positively selected site, PSS) if it could be identified in at least two of the four methods used.

## Results and discussion

### MHC sequence characterisation and copy number variation

**Fig. 1:**
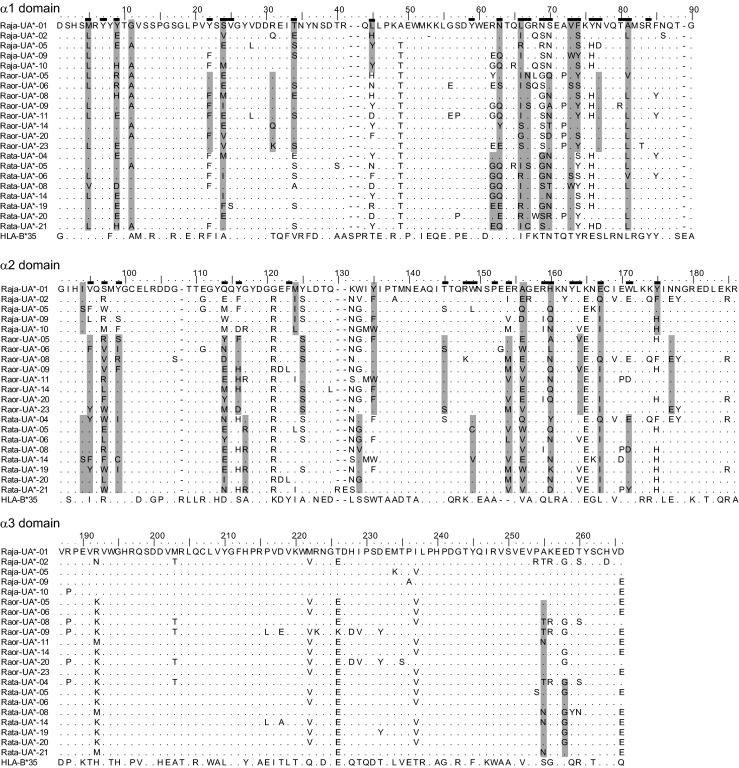
Amino acid alignment of selected MHC class I variants from *R. japonica* (*Raja*), *R. ornativentris* (*Raor*) and *R. t. tagoi* (*Rata*). Alignment is divided into the three domains studied. Codon sites shaded in *grey* are detected as under positive selection independently in each species by at least two of four methods used (omegaMap, FEL, REL and MEME); referred to as positively selected sites (*PSS*). Putative PBR sites inferred from humans (Bjorkman et al. 1987; Saper et al. 1991) are indicated by *black bars above the leading sequence*. Amino acid sequence for a well-characterised HLA allele, HLA-B*35, is also included (HLA-B*35:01:01:01, IMGT/HLA acc. no. HLA00237)

**Fig. 2:**
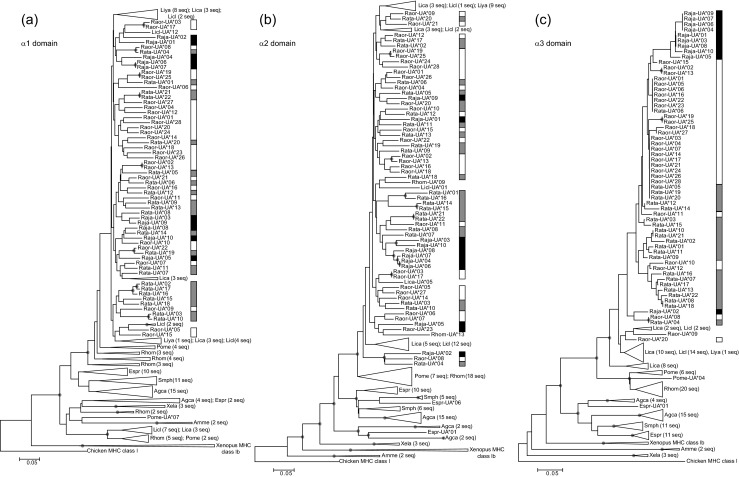
Phylogenetic relationships between MHC I variants identified in *R. japonica* (*Raja*, *black bar*), *R. ornativentris* (*Raor*, *white bar*) and *R. t. tagoi* (*Rata*, *grey bar*) and other amphibians (neighbour-joining method). We constructed phylogenies independently for the α1 domain (**a**), α2 domain (**b**) and α3 domain (**c**). *Shaded circles* indicate nodes with bootstrap support >70 %. Accession numbers for sequences from other species include *Gallus gallus* MHC class I (L28958.2); *Xenopus* MHC class Ib (NM_001247995, FJ589643, L20726); MHC class Ia: *X. laevis* Xela (AF185580, AF185582, AF185583), *Ambystoma mexicanum* Amme (U83137, U83138), *Rhacophorus omeimontis* Rhom and *Polypedates megacephalus* Pome (KC261637–KC261663), *Agalychnis callidryas* Agca, *Espadarana prosoblepon* Espr, *Smilisca phaeota* Smph, *Lithobates catesbeianus* Lica, *L. clamitans* Licl and *L. yavapaiensis* Liya (JQ679312–JQ679390)

We identified a total of 10, 28 and 22 MHC class I sequences (referred to here as variants) from *R. japonica*, *R. ornativentris* and *R. t. tagoi*, respectively (Table 1); these variants are designated *Raja*-UA*01–10, *Raor*-UA*01–28 and *Rata*-UA*01–22 (Genbank accession nos. KX100486–KX100545). These newly characterised variants were all 780 bp (260 codons) in length (Fig. 1), with the exception of *Raja*-UA*02 (259 codons) and *Rata*-UA*21 and 22 (262 codons). All variants likely belong to classical class I loci as they were amplified from RNA, clustered phylogenetically with other classical class I sequences (Fig. 2) and had no premature stop codons or aberrant indels. There were no MHC class I variants shared between the species studied. Within *R. japonica*, many variants were shared across individuals, especially *Raja*-UA*01 (amplified from six of seven individuals) and *Raja*-UA*03 and *Raja*-UA*04 (amplified from four of seven individuals each); this could be attributed to the lower effective population size of this island population studied (T. Igawa, unpublished data). In contrast, there were no shared MHC class I variants between the seven *R. t. tagoi* individuals studied and only one shared variant (*Raor*-UA*06) between two *R. ornativentris* individuals (Supplementary material 1A). Our initial characterisation of MHC class I sequences in these three Japanese frog species provides the framework for future studies of genetic diversity of these abundant species across their broad habitat range across Japan.

**Table 1:** Summary of MHC class I variants, genetic divergence and codon-based *Z* tests for selection in the three Japanese *Rana* species

Species	No. of variants (total)	No. of variants (per individual)	Total no. of segregating sites^a^		Range of divergence within individual		Mean distance (nucleotide, amino acid)			*Z* tests for selection for α1 and α2 domains combined (*Z* statistic)		
			Nucleotide	Amino acid	Nucleotide	Amino acid	α1	α2	α3	Neutrality	Purifying	Positive
*R. japonica*	10	2–5	136	78	0.012–0.115	0.031–0.212	0.064, 0.135	0.080, 0.155	0.024, 0.045	4.199*	−4.204 n.s.	4.219**
*R. ornativentris*	28	3–5	192	94	0.063–0.104	0.108–0.173	0.067, 0.158	0.067, 0.122	0.035, 0.048	3.446*	−3.422 n.s.	3.372*
*R. t. tagoi*	22	2–5	167	83	0.050–0.101	0.100–0.169	0.081, 0.149	0.081, 0.152	0.036, 0.060	3.808**	−3.808 n.s.	3.920**

*p < 0.01, **p < 0.0001, n.s.: *p* > 0.05

^a^Excludes insertions or deletions

**Fig. 3:**
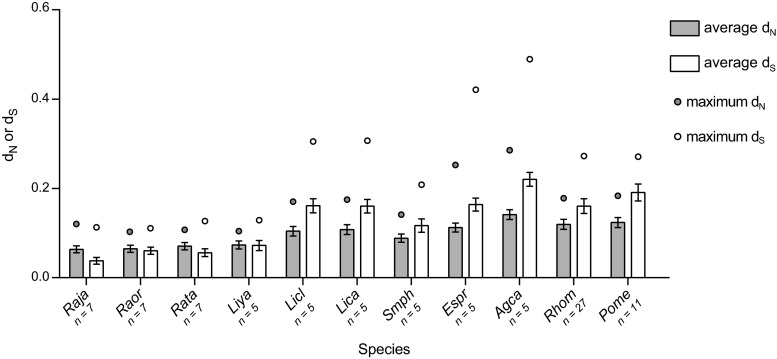
Average number of non-synonymous differences per non-synonymous site (*d*_N_, *grey bars*) or synonymous differences per synonymous site (*d*_S_, *white bars*) in MHC class I of various frog species using the Nei–Gojobori model (proportion) with 1000 bootstrap replicates. *Error bars* represent standard error estimates; *circles* represent maximum *d*_N_ or *d*_S_ from a pair of variants. Sequences from other anuran species were characterised by Kiemnec-Tyburczy et al (2012) and Zhao et al (2013)

Two to five sequences were characterised from each individual (Supplementary material 1A); it is likely we have underestimated the total number variants per individual due to conservative approaches for confirming MHC variants, similar to Zhao et al (2013), as well as the limited number of sequenced clones. Future studies of additional individuals and populations will likely uncover more MHC class I variation in these expansive and unthreatened species. The sequence divergence within individuals at the nucleotide and amino acid levels (intra-individual variation) was similar amongst the three study species (Table 1 and Supplementary material 1A). The variation is also comparable to the six frog species (*n* = 5 individuals per species) studied by Kiemnec-Tyburczy et al (2012), although higher amino acid divergence was detected in some *A. callidryas* (0.451) and *E. prosoblepon* (0.336) individuals. This could be related to the unusually low levels of per-site differences at MHC class I in the three Japanese *Rana* species, especially the average synonymous differences per synonymous site (*d*_S_; Fig. 3). Subsequently, the resulting *d*_N_/*d*_S_ ratio (or *ω*) of MHC class I from *R. japonica* (*ω* = 1.67), *R. ornativentris* (*ω* = 1.07) and *R. t. tagoi* (*ω* = 1.26) is higher than that of previously studied frogs (*ω* = 0.64–0.76), with the exception of *L. yavapaiensis* (*ω* = 1.02).

Since up to five variants were found in each individual, up to three loci were amplified; this aligns with recent anuran studies that have found MHC class I copy number variation, including up to three loci in some Ranidae species (Kiemnec-Tyburczy et al. 2012; Zhao et al. 2013). As with MHC studies in other non-model species, it remains difficult to completely map out the total number of MHC loci in these species without the availability of whole genome sequences. Comparison of maximum synonymous divergence between assumed paralogous sequences (that is, the most divergent sequence pairs) showed lower maximum *d*_S_ in the three focal species (Fig. 3), indicative of a more recent gene duplication relative to other frogs studied.

The total number of variants in the studied cohort of *R. japonica* was lower than that of the other two focal species, likely due to the population history or kin relationships. Despite the lower total number of variants and segregating sites in *R. japonica*, this cohort had a higher maximum intra-individual sequence variation and comparable overall mean sequence distance relative to the other two species (Table 1). This suggests that the sufficient sequence diversity could offset the low variant diversity in the *R. japonica* Etajima cohort. Further studies of more extant populations of *R. japonica* may reveal whether there is additional sequence variation in this species. The α1 and α2 domains (exons 2 and 3, respectively) in all species had higher mean genetic distances than the α3 domain (exon 4). This is consistent with the theory that the α3 domain is more conserved and functionally constrained whilst the α1 and α2 domains have high genetic variation for diverse peptide recognition capacity (Colombani 1990).

### Phylogenetic relationships and trans-species polymorphism

We constructed phylogenetic trees of each domain of MHC class I from the three Japanese *Rana* species along with previously studied amphibians. Within the three focal species, we found no species-specific phylogenetic clustering at the α1 and α2 domains, whereas there were possible monophyletic groups in the α3 domain, especially for *R. japonica* (Fig. 2 and Supplementary material 1B). Whilst there was generally weak bootstrap support across most branches, which restricts us from making strong conclusions regarding trans-species polymorphism, this polyphyletic pattern is consistent with previous frog MHC class I studied by Kiemnec-Tyburczy et al (2012) and Zhao et al (2013). For α1 and α2 domains, we observed that some variants from other Ranidae species clustered together with Japanese *Rana* (Fig. 2a, b); in addition, within the α2 domain, there was clustering with sequences from more extant Rhacophoridae species. Despite the low branch support, there were three variants, one from each of the focal species (*Raja*-UA*02, *Raor*-UA*08 and *Rata*-UA*04), that formed a distinct basal polyphyletic group in the α2 and α3 domains (Fig. 2 and Supplementary material 1B). Although these three variants were only identified in one individual per species, they could be derived from a more ancient MHC locus that has been maintained since the three Japanese species diverged, adding support for trans-species polymorphism.

### Evidence of recombination

Recombination is one of the main mechanisms proposed as evolutionary drivers of high diversity in MHC and generation of novel alleles (Carrington 1999). We tested the full-length MHC class I sequences of each of the three focal species (covering exons 2–4) and found multiple signals of recombination. Using GARD, three to five recombination breakpoints were identified in each species (Table 2), some of which were located near exon boundaries located at nucleotide positions 261 and 546. This supports recombination through the mechanism of exon shuffling, which is commonly found in lower vertebrates such as fishes and amphibians (Bos and Waldman 2006; Wang et al. 2010; Zhao et al. 2013). The RDP programme was more conservative in detecting recombination, identifying only one and three breakpoints in *R. ornativentris* and *R. t. tagoi*, respectively (Table 2); many of these breakpoints were concordant (within a 20-bp window) with those detected by GARD (Table 2). We have found evidence of recombination acting on MHC class I sequences of the three focal species, and such recombination was accounted for in subsequent tests for detecting codon-based selection.

**Table 2:** Identification of recombination breakpoints in MHC class I variants within each of the three *Rana* species studied

Species	GARD: nucleotide breakpoints	RDP		
		Nucleotide breakpoint	Recombinant variant(s)	Potential major/minor parent variants
*R. japonica*	92, 329, 493	None	–	–
*R. ornativentris*	82, 362, 516	510	*Raor*-UA*01 and 04	Unknown/*Raor*-UA*05
*R. t. tagoi*	127, 248, 393, 501, 641	194 and 406	*Rata*-UA*19	*Rata*-UA*20/*Rata*-UA*04
		510	*Rata*-UA*17 and 18	*Rata*-UA*06/*Rata*-UA*08

### Selection acting on MHC sequences

At the amino acid level, balancing selection can maintain alleles and variation for evolutionarily long periods and can even retain similar or identical alleles between diverged species (Klein et al. 1998), which we observed in the phylogenetic relationships (Fig. 2). Additional support for selection in MHC class I genes of the three focal *Rana* species came from identification of (1) specific codon sites under positive selection and (2) overall selection at the sequence level.

The total number of sites identified to be under positive selection varied between the four codon-based methods used and the three study species (Supplementary material 1C). Therefore, we identified PSS under the criterion of detection by at least two of the four methods and found a total of 18, 30 and 28 PSS in *R. japonica*, *R. ornativentris* and *R. t. tagoi*, respectively (Table 3 and Fig. 1; Supplementary material 1C). Overall, across the three focal species, 18 PSS was defined in the α1 domain and 21 PSS defined in the α2 domain. Of these 39 PSS from Japanese *Rana*, a majority were identical sites to those defined in other frogs (27 sites) by Kiemnec-Tyburczy et al (2012) and Zhao et al (2013), as well as PBR sites in human leukocyte antigen (HLA; 28 sites) determined by Bjorkman et al (1987) and Saper et al (1991) (Table 3). The previous anuran studies combined more than one species to identify PSS, not considering differences in selection between species, whilst we found just as many PSS independently in the three Japanese *Rana* species; this includes some selected sites in the α2 domain that may be species-specific (codon sites 124, 125, 133 and 177; Fig. 1 and Table 3). Nonetheless, the many PSS that are in agreement with other anurans and humans are likely to be polymorphic sites that are functionally important for antigen binding and maintained across vertebrate evolutionary history. In the α3 domain, only two PSS were identified across the three study species, re-emphasising that this domain has been conserved at the sequence level and has not been under the impact of diversifying selection.

**Table 3:** Codon sites predicted to be under positive selection in the three Japanese *Rana* species, referred to as positively selected sites

Species	Codon sites predicted to be under positive selection																																								
	α1 domain																		α2 domain																					α3 domain	
	5	9	11	22	24	31	34	45	62	63	66	67	69	70	73	74	77	81	94	95	97	99	114	116	117	124	125	133	135	145	149	154	156	160	164	167	171	175	177	255	258
*R. japonica*	˄	˄	˅		˅		˅	˄		˄	˄				˅	˄		˅	˄							˄			˄				˄	˄		˄		˄			
*R. ornativentris*	˅	X	X	˄	˄	˅	X			X	X	˅		X	X	X	˄	X		X	X	˅	˅	X			˅		˅	˅		˄	X	X	˅	˄			˄	˅	
*R. t. tagoi*	˄	X	X		˅				˄	˅	˄	X	˄	X	X			X	˄	˄	˅	˄	˅		X			˄			˄	X	X	˄		˅	˄			˄	X
Human^a^	Y	Y		Y	Y		Y	Y		Y	Y	Y	Y	Y	Y	Y	Y	Y		Y	Y	Y	Y	Y						Y	Y	Y		Y	Y	Y	Y	Y		–	–
Other frogs^b^	–	Y	Y		Y		Y	Y	Y	Y	Y	Y	Y	Y	Y			Y	Y	Y	Y	Y	Y	Y	Y				Y	Y		Y	Y	Y		Y		Y			Y

Sites are considered to be under positive selection if detected by at least two (˄), three (˅) or all (X) of the four methods used (omegaMap, FEL, REL and MEME). Many PSS were also deduced or predicted to be selected sites (Y) in humans or other frogs. Codon sites are based on the alignment in Fig. 1. ‘–’ indicates the site was not tested

^a^Deduced PBR sites from HLA based on Bjorkman et al (1987) and Saper et al (1991)

^b^Predicted selected sites in other frog species based on Kiemnec-Tyburczy et al (2012) and Zhao et al (2013)

Selection tests over the entire sequence showed evidence of positive selection (*Z* = 1.950–4.024, *p* < 0.05) and rejection of neutrality and purifying selection (Supplementary material 1D). We further examined selection at each domain and found that the α1 and α2 domains showed evidence supporting positive selection (*p* < 0.01) and against neutrality and purifying selection (Table 1 and Supplementary material 1D). We also identified similar support for positive selection when using PARRIS (Supplementary material 1D). Meanwhile, the α3 domain showed no sequence-wide positive selection (*Z* = −2.373–0.0431, *p* > 0.40), and for the particular case for *R. ornativentris*, there was even evidence of purifying selection at this domain (*Z* = 2.335, *p* = 0.011; Supplementary material 1D). These sequence-wide selection tests contribute to supporting the functional roles of the α1 and α2 domains and the conserved nature of the α3 domain.

Interestingly, Kiemnec-Tyburczy et al (2012) used one-tailed *Z* tests and found no evidence of selection in any domain in any of the six anuran species that were studied, whilst PARRIS could detect positive selection. We confirmed this pattern in those sequences as well as sequences isolated from Rhacophoridae species by Zhao et al (2013), supporting that the positively selected sites in MHC class I are masked by the large number of neutrally evolving sites. The inability to detect overall neutrality at MHC class I in the three focal study species here could be attributed to either lower per-site synonymous differences and/or stronger selection pressure acting on the molecule. The small extent of synonymous differences (*d*_S_) of MHC class I within Japanese *Rana* species (Fig. 3) could be explained by both within- and between-loci divergence. Low per-site differences in variants (within-loci) could be attributed to severe bottlenecks associated with hypothesised shrinkage of ancestral frog populations during migration from the Asian continent to the Japanese archipelago, perhaps restricted through land bridges (Igawa et al. 2006; Komaki et al. 2015). For between-loci divergence, lower maximum synonymous differences (Fig. 3) in Japanese *Rana* species support a more recent duplication of loci and subsequent diversification of paralogous variants. The low *d*_S_ results in a positive and inflated average *ω* (*d*_N_/*d*_S_) relative to other anurans, which suggests that the selection pressure acting on MHC class I in Japanese *Rana* species may be stronger than in other frog species studied. More specifically, the strong positive selection pressure could be targeted at peptide binding sites since we found a similar number of PSS in the three focal species compared to other frogs.

## Conclusion

Here, we have characterised the MHC class I classical variation in three Japanese true *Rana* frog species, with high diversity found in these common unthreatened species. We have identified evidence supporting three major mechanisms that drive high MHC diversity. Signatures of recombination, balancing selection and recent gene duplication found in Japanese *Rana* MHC class I are similar to those of previous anuran studies. This study is the springboard for investigating genetic variation at other adaptive loci in Japanese amphibians, which will help to better understand the polygenetic basis of resistance to infectious diseases such as chytridiomycosis.

## Electronic supplementary material

Below is the link to the electronic supplementary material.

ESM 1 (PDF 534 kb)
