# Supplementary material for: Characterisation of major histocompatibility complex class I genes in Japanese Ranidae frogs
Source: Immunogenetics. 2016 Jul 14;68(10):797–806. doi: 10.1007/s00251-016-0934-x (PMC5056945; doi:10.1007/s00251-016-0934-x)
Supplement: Supplementary file 1 — (PDF 534 kb) [file 251_2016_934_MOESM1_ESM.pdf]

Supplementary material

A. MHC class I variants identified in each individual and range of pairwise nucleotide (ncl) or amino acid (aa) difference within individual. Variants in bold were observed in more than one individual.

Individual number	*R. japonica*			*R. ornativentris*			*R. tagoi*		
	Variants	*ncl*	*aa*	*Variants*	*ncl*	*aa*	*Variants*	*ncl*	*aa*
1	*Raja*-UA***01**, 07	0.040	0.088	*Raor*-UA*01, 02, 26	0.044-0.063	0.081-0.116	*Rata*-UA*01, 07, 08, 09	0.050-0.079	0.096-0.138
2	*Raja*-UA***01**, **03**, **05**, 08, 09	0.033-0.076	0.073-0.146	*Raor*-UA***06**, 07, 08, 09, 25	0.050-0.104	0.092-0.173	*Rata*-UA*02, 03, 10	0.028-0.058	0.050-0.108
3	*Raja*-UA***01**, **03**, **05**, 10	0.038-0.076	0.077-0.146	*Raor*-UA*16, 17, 22, 23, 24	0.044-0.077	0.081-0.119	*Rata*-UA*17, 18, 22	0.043-0.058	0.088-0.104
4	*Raja*-UA***01**, 02, **03**, **04**	0.046-0.115	0.097-0.212	*Raor*-UA***06**, 14, 15, 27, 28	0.055-0.078	0.092-0.131	*Rata*-UA*11, 12	0.050	0.100
5	*Raja*-UA***01**, **04**, **05**	0.047-0.065	0.112-0.131	*Raor*-UA*13, 18, 19, 20, 21	0.045-0.069	0.088-0.138	*Rata*-UA*13, 14	0.068	0.131
6	*Raja*-UA***04**, 06	0.012	0.031	*Raor*-UA*03, 04, 05	0.058-0.065	0.096-0.108	*Rata*-UA*04, 05, 06, 19, 20	0.053-0.101	0.100-0.169
7	*Raja*-UA***01**, **03**, **04**, **05**	0.046-0.076	0.096-0.146	*Raor*-UA*10, 11, 12	0.058-0.076	0.108-0.150	*Rata*-UA*15, 16, 21	0.060-0.072	0.104-0.131

B. Phylogenetic relationships between MHC class I sequences from *R. japonica* (black bar), *R. ornativentris* (white bar), and *R. t. tagoi* (grey bar) compared to other amphibians, using maximum likelihood method. Trees were constructed independently for (a) α1 domain, (b) α2 domain, and (c) α3 domain. Shaded circles indicate nodes with bootstrap support > 70%. Inferred relationships are comparable to those determined by neighbour-joining method in Figure 2. The basal *Rana* clade (containing *Raja*–UA*02, *Raor*–UA*08 and *Rata*–UA*04) is present in α2 and α3 trees. Accession numbers for sequences from other species listed in Figure 2.

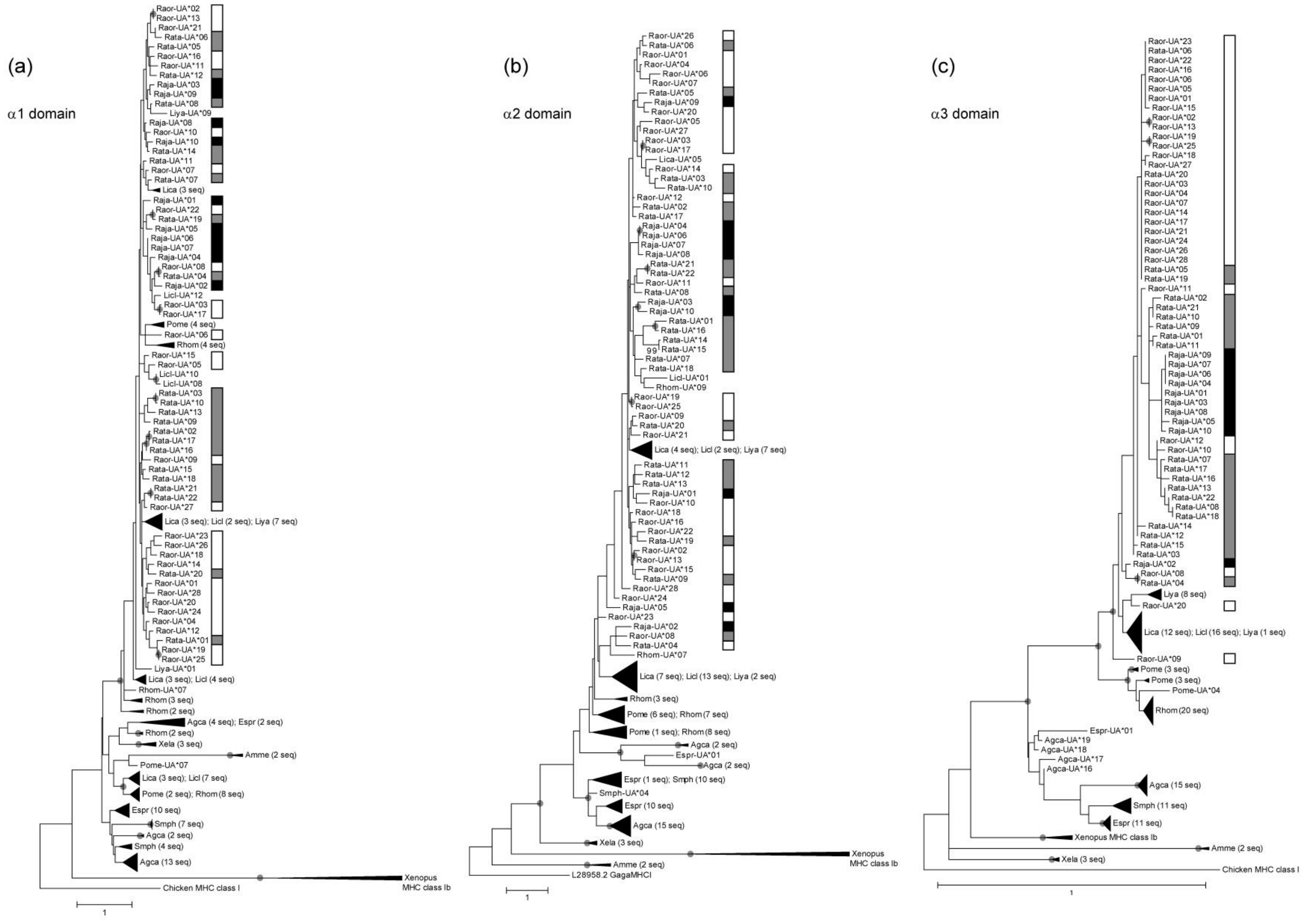

C. Codon sites predicted to be under positive selection in the three Japanese *Rana* species, as detected by each of the four methods omegaMap (OM), FEL, REL, and MEME.

Site	*R. japonica*					*R. ornativentris*					*R. t. tagoi*				
	OM	FEL	REL	MEME	Total	OM	FEL	REL	MEME	Total	OM	FEL	REL	MEME	Total
5	y		y		2		y	y	y	3	y		y		2
6					0					0	y				1
9	y		y		2	y	y	y	y	4	y	y	y		4
11	y	y	y		3	y	y	y	y	4	y	y	y	y	4
15	y				1					0					0
22					0		y	y		2					0
24	y		y	y	3	y			y	2	y		y	y	3
25					0					0	y				1
28					0	y				1					0
31					0	y	y	y		3					0
34	y	y	y		3	y	y	y	y	4	y				1
44	y				1					0					0
45	y		y		2	y				1	y				1
49	y				1					0					0
62	y				1	y				1	y			y	2
63	y			y	2	y	y	y	y	4	y		y	y	3
66	y		y		2	y	y	y	y	4	y			y	2
67	y				1	y	y	y		3	y	y	y	y	4
68					0	y				1					0
69	y				1	y				1	y			y	2
70					0	y	y	y	y	4	y	y	y	y	4
72					0			y		1	y				1
73	y		y	y	3	y	y	y	y	4	y	y	y	y	4
74	y		y		2	y	y	y	y	4					0
76	y				1			y		1					0
77	y				1	y		y		2					0
81	y		y	y	3	y	y	y	y	4	y	y	y	y	4
83					0	y				1					0
85					0			y		1					0
94	y			y	2					0	y			y	2
95	y				1	y	y	y	y	4	y			y	2
97	y				1	y	y	y	y	4	y		y	y	3
99	y				1	y		y	y	3	y			y	2
114	y				1	y		y	y	3	y		y	y	3
116	y				1	y	y	y	y	4	y				1
117	y				1					0	y	y	y	y	4
123					0	y				1					0
124	y		y		2					0	y				1
125					0		y	y	y	3					0
129					0	y				1					0
132	y				1					0	y				1
133	y				1			y		1	y	y			2
134					0	y				1					0
135	y		y		2	y		y	y	3	y				1
145	y				1	y	y	y	y	4			y		1
149					0					0	y			y	2
154	y				1	y		y		2	y	y	y	y	4
156	y		y		2	y	y	y	y	4	y	y	y	y	4
159	y				1					0					0
160	y		y		2	y	y	y	y	4	y		y		2
164	y				1		y	y	y	3	y	y	y	y	4
165	y				1					0					0
167	y			y	2	y		y	y	3	y		y	y	3
169	y				1					0	y				1
171	y				1	y				1	y			y	2
175	y		y		2			y		0	y				1
177	y				1			y	y	2	y				1
185	y				1			y		1					0
188	y				1			y		1	y				1
192	y				1					0					0
203	y				1					0	y				1
226	y				1					0					0
237	y				1					0	y				1
255					0	y		y	y	3	y			y	2
258					0			y		1	y	y	y	y	4
260	y				1	y				1	y				1
266	y				1					0					0
SUM	50	2	15	6	18	33	21	37	25	30	45	12	19	25	28

D. Codon-based z-tests for selection (neutrality, purifying and positive selection) for each domain and globally in MHC class I; results presented as Z-statistic (p-value), Z-statistics with significant values ($p < 0.05$) are in bold; positive selection was also tested using PARRIS implemented in the Datamonkey webserver, with results presented as [p-value].

Species	Neutrality					Purifying					Positive				
	Overall	α1	α2	α1 + α2	α3	Overall	α1	α2	α1 + α2	α3	Overall	α1	α2	α1 + α2	α3
R. japonica	**4.030**<br>(<0.001)	**3.536**<br>(<0.001)	**2.472**<br>(0.015)	**4.199**<br>(<0.001)	0.042<br>(0.966)	-3.959<br>(1.000)	-3.503<br>(1.000)	-2.497<br>(1.000)	-4.204<br>(1.000)	-0.043<br>(1.000)	**4.024**<br>(<0.001)<br>[<0.001]	**3.493**<br>(<0.001)<br>[0.001]	**2.449**<br>(0.008)<br>[0.145]	**4.219**<br>(<0.001)<br>[<0.001]	0.0431<br>(0.4828)<br>[0.922]
R. ornativentris	**2.031**<br>(0.044)	**2.808**<br>(0.006)	**2.010**<br>(0.047)	**3.446**<br>(0.001)	**-2.290**<br>(0.024)	-1.961<br>(1.000)	-1.991<br>(1.000)	-2.809<br>(1.000)	-3.422<br>(1.000).	**2.335**<br>(0.011)	**1.950**<br>(0.027)<br>[<0.001]	**2.880**<br>(0.002)<br>[<0.001]	**1.960**<br>(0.026)<br>[<0.001]	**3.372**<br>(0.001)<br>[<0.001]	-2.373<br>(1.000)<br>[1.000]
R. t. tagoi	**3.186**<br>(0.002)	**2.057**<br>(0.042)	**3.443**<br>(0.001)	**3.808**<br>(<0.001)	-1.097<br>(0.275)	-3.198<br>(1.000)	-2.057<br>(1.000)	-3.443<br>(1.000)	-3.808<br>(1.000)	1.106<br>(0.135)	**3.176**<br>(0.001)<br>[<0.001]	**2.057**<br>(0.021)<br>[0.006]	**3.363**<br>(0.001)<br>[<0.001]	**3.920**<br>(<0.001)<br>[<0.001]	-1.128<br>(1.000)<br>[1.000]
